# Immunogenicity of Live Attenuated *B. pertussis* BPZE1 Producing the Universal Influenza Vaccine Candidate M2e

**DOI:** 10.1371/journal.pone.0059198

**Published:** 2013-03-19

**Authors:** Hana Kammoun, Xavier Roux, Dominique Raze, Anne-Sophie Debrie, Marina De Filette, Tine Ysenbaert, Nathalie Mielcarek, Xavier Saelens, Walter Fiers, Camille Locht

**Affiliations:** 1 Inserm U1019, Lille, France; 2 CNRS UMR 8204, Lille, France; 3 Institut Pasteur de Lille, Center for Infection and Immunity of Lille, Lille, France; 4 University Lille Nord de France, Lille, France; 5 Department for Molecular Biomedical Research, VIB, Ghent, Belgium; 6 Department of Biomedical Molecular Biology, Ghent University, Ghent, Belgium; Universidad Nacional de La Plata, Argentina

## Abstract

**Background:**

Intranasal delivery of vaccines directed against respiratory pathogens is an attractive alternative to parenteral administration. However, using this delivery route for inactivated vaccines usually requires the use of potent mucosal adjuvants, and no such adjuvant has yet been approved for human use.

**Methodology/Principal Findings:**

We have developed a live attenuated *Bordetella pertussis* vaccine, called BPZE1, and show here that it can be used to present the universal influenza virus epitope M2e to the mouse respiratory tract to prime for protective immunity against viral challenge. Three copies of M2e were genetically fused to the N-terminal domain of filamentous hemagglutinin (FHA) and produced in recombinant BPZE1 derivatives in the presence or absence of endogenous full-length FHA. Only in the absence of FHA intranasal administration of the recombinant BPZE1 derivative induced antibody responses to M2e and effectively primed BALB/c mice for protection against influenza virus-induced mortality and reduced the viral load after challenge. Strong M2e-specific antibody responses and protection were observed after a single nasal administration with the recombinant BPZE1 derivative, followed by a single administration of M2e linked to a virus-like particle without adjuvant, whereas priming alone with the vaccine strain did not protect.

**Conclusions/Significance:**

Using recombinant FHA-3M2e-producing BPZE1 derivatives for priming and the universal influenza M2e peptide linked to virus-like particles for boosting may constitute a promising approach for needle-free and adjuvant-free nasal vaccination against influenza.

## Introduction

Respiratory pathogens are the leading cause of global deaths from infectious diseases [1]. Vaccines against some respiratory pathogens are available, and most often these vaccines are administered by needle injection. However, intranasal (i.n.), and more generally mucosal vaccination can be an effective way to immunize against respiratory infections. This mode of vaccine delivery has a number of advantages over conventional vaccination [2], including needle-free administrations of vaccines and the potential of inducing immunity at mucosal sites, the entry port of respiratory pathogens. However, most antigens are poorly immunogenic when applied by the nasal route, and potent adjuvants are often needed. Examples of such adjuvants include genetically detoxified cholera toxin and the related *Escherichia coli* heat-labile enterotoxin, which are among the most potent mucosal adjuvants known. However, their i.n. application in the formulation of an influenza vaccine has raised safety concerns as it resulted in unacceptable adverse events, such as Bell’s palsy [3]. As an alternative way to effectively deliver antigens to the respiratory mucosa, live attenuated vectors have also been explored. Live attenuated influenza virus has been successfully tested in humans, including infants, and was found to be safe and able to induce protective immunity after a single i.n. application [4].

We have recently developed a live attenuated *Bordetella pertussis* vaccine candidate, initially designed to protect against whooping cough. This vaccine candidate, named BPZE1, was generated by the genetic removal or inactivation of three major *B. pertussis* toxins [5]. In preclinical models, it showed an excellent safety profile, including in severely immuno-compromized animals [6]. Despite its strong attenuation, BPZE1 is able to colonize the respiratory tract and to induce strong and long-lasting protective immunity, even in 1-week-old mice [7]. These properties and the documented genetic stability of the strain [8] have allowed BPZE1 to be downgraded from biosafety level 2 to level 1 and to undergo first-in-man clinical trials (ClinicalTrials.gov NCT01188512).

Furthermore, BPZE1 displays potent anti-inflammatory properties and was found to protect against experimental allergic asthma [9], [10] and against mortality induced by highly pathogenic influenza viruses [11] by dampening the virus-induced cytokine storm.

We have previously shown that recombinant *B. pertussis* strains can also be used as multivalent vaccine candidates able to protect simultaneously against both pertussis and heterologous pathogens [12]–[17]. Here, we used a truncated form of filamentous hemagglutinin (FHA), named Fha44, containing its secretion determinant to export the 23-amino-acid extracellular domain of the influenza A virus matrix protein M2 (M2e) from BPZE1. M2e is remarkably well conserved among human influenza A virus isolates and has been proposed as a universal influenza vaccine antigen [18]–[21]. Fused to the hepatitis B virus core protein as a virus-like particle (VLP) M2e conferred protection against a lethal influenza A virus challenge in the mouse model [19].

In a previous study, BPZE1 has been engineered to produce one, two or three copies of M2e fused to full-length FHA [17]. However, secretion efficiency decreased with the numbers of M2e copies, and the hybrid protein containing 3 copies of M2e was barely detectable in the culture supernatant of the recombinant strain. Antibody responses to M2e in mice were detectable, but weak, even after three i.n. administrations of high doses of the recombinant strain. Since Fha44 is more efficiently secreted than full-length FHA [22], we used this protein as a carrier for M2e in order to optimize secretion efficiency and immunogenicity. We show here that the BPZE1 derivative producing Fha44-M2e is able to induce an immune response only in the absence of full-length FHA. Furthermore, i.n. administration of FHA-deficient BPZE1 producing Fha44-M2e effectively primed the immune system for a heterologous boost with an i.n. administered M2e-VLP vaccine. This mucosal prime-boost regimen, in the absence of adjuvants, protected mice against a lethal challenge with influenza A virus, whereas priming alone with the vaccine strain did not protect.

## Results

### Construction of BPZM2e

To express the extracellular domain of the M2 protein (M2e) of influenza A virus in BPZE1, we used Fha44 as carrier. Fha44 is the 80-kDa N-terminal fragment of FHA and is better secreted by *B. pertussis* than full-length FHA [22]. Three copies of the M2e-coding sequence were fused to the Fha44-coding sequence. The M2e sequence differs from the original sequence by the substitution of the two cysteines (Cys-16 and Cys-18) for serines in order to optimize secretion by Fha44, as we have previously shown that the formation of disulfide bonds may hamper Fha4-mediated secretion [23]. The construct was inserted into the BPZE1 chromosome at the *dnt* locus by allelic exchange, placing the transgene under the control of the *dnt* promoter. This yielded the recombinant strain BPZM2e.

**Figure 1:**
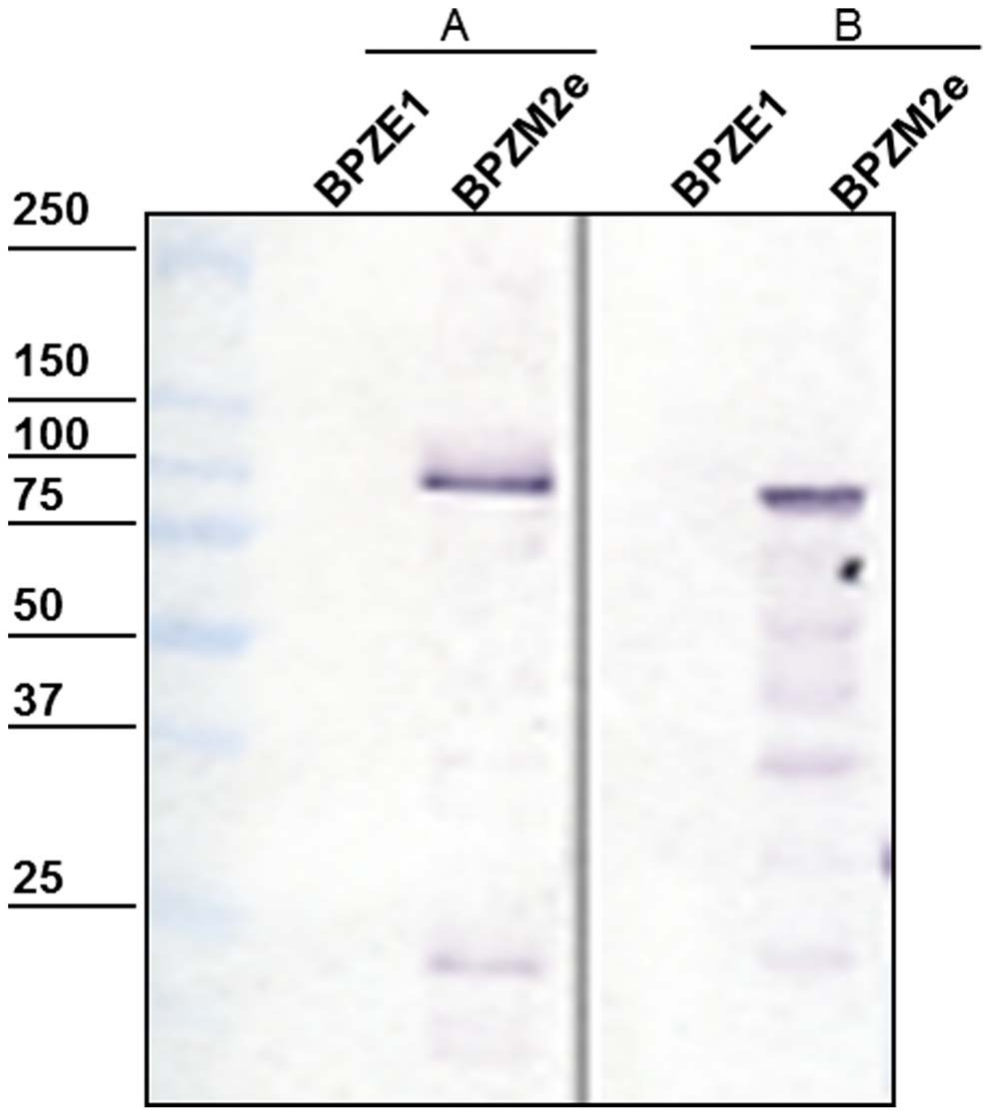
Immunoblot analysis of BPZM2e.

Crude culture supernatants and whole cell extracts of BPZE1 and BPZM2e were examined by immunoblot analysis using an anti-M2e monoclonal antibody. As shown in Figure 1, a 94-kDa band, corresponding to the expected size of the Fha44-3M2e chimeric protein, was detected in the culture supernatant of the recombinant strain. A protein of similar size reactive with the anti-M2e antibody was also detected in the whole cell extracts. This observation indicates that the chimeric protein was secreted from the recombinant strain and was also associated with BPZM2e cells.

### Lung Colonization and Immunogenicity of BPZM2e

**Figure 2:**
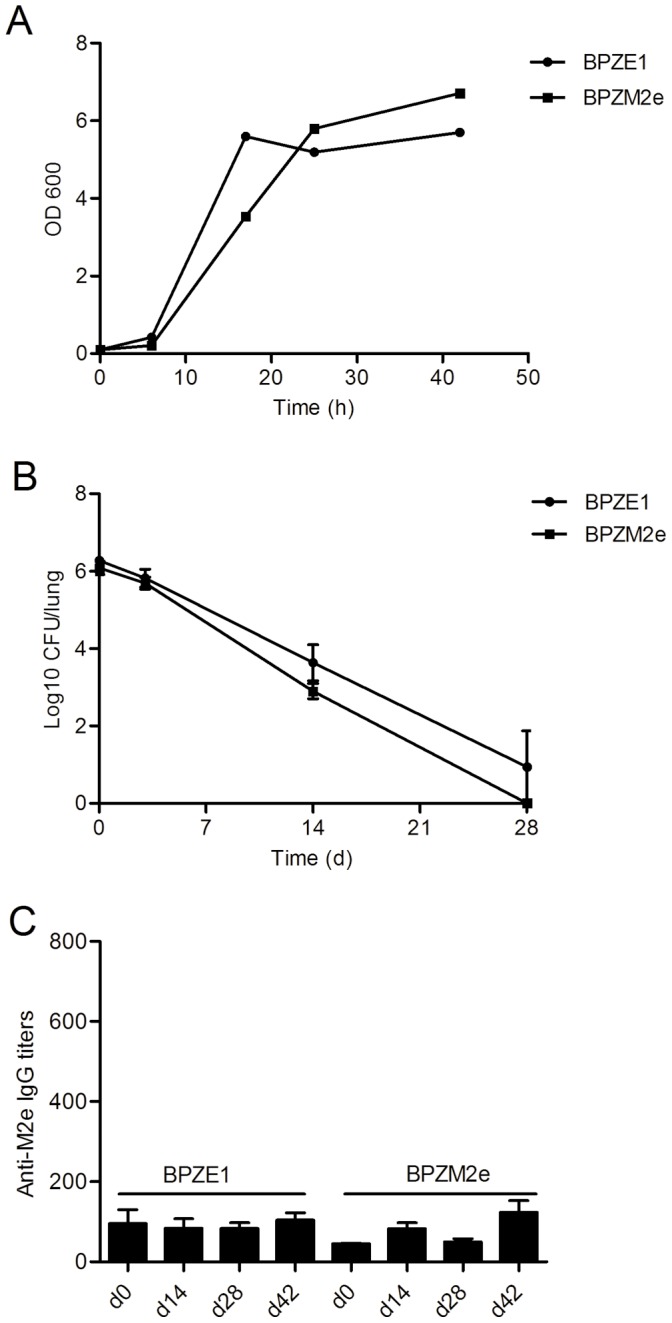
Growth curves, colonization capacity and immunogenicity of BPZM2e. (A) The growth curves of BPZE1 (closed circles) and BPZM2e (closed squares) in Stainer Scholte medium were determined by OD measurements at 600 nm over the indicated periods of time. (B) BALB/c mice were inoculated i.n. with 10^6 ^CFU of BPZE1 or recombinant BPZM2e. Bacterial colonization of the lungs by BPZE1 (closed circles) and BPZM2e (closed squares) was measured in CFU over the indicated periods of time following i.n. administration. (C) BALB/c mice were i.n. immunized twice at a 4-week interval with 10^7 ^CFU of BPZE1 or recombinant BPZM2e as indicated. Sera were collected at indicated time points after the first immunization, and serum IgG responses to M2e were determined by an M2e-peptide ELISA.

To evaluate the general fitness of BPZM2e its *in vitro* growth kinetics was compared to that of the parent strain BPZE1. As shown in Figure 2A, there was no significant difference between the two strains, indicating that the general bacterial fitness was not impaired by the production of Fha44-3M2e.

To study the ability of BPZM2e to colonize the murine respiratory tract, BALB/c mice were inoculated i.n. with 10^6^ CFU of BPZM2e or with non-recombinant BPZE1, and their colonization profiles were compared. The colonization profile of BPZM2e was indistinguishable from that of the corresponding parental strain BPZE1 (Figure 2B), indicating that the production of Fha44-3M2e does not alter the ability of the bacteria to colonize the lungs of mice.

The antibody responses to the M2e peptide were examined by ELISA at different time points after administration of 10^6^ CFU of BPZM2e to the mice. However, no M2e-specific antibodies were detected in the serum at any time point (data not shown). The BPZM2e dose was then increased ten-fold, and BALB/c mice were i.n. immunized twice with a 4-week interval with 10^7^ CFU of BPZE1 or recombinant BPZM2e. Sera were collected at 2 and 4 weeks after the first immunization, and 2 weeks after the last immunization to measure the systemic anti-M2e IgG response. Again, no significant antibody response to M2e was detected in the sera (Figure 2C).

### Inactivation of FHA and Characterization of BPZM2e-ΔFHA

We hypothesized that the endogenous FHA in BPZM2e may perhaps act as an immunodominant antigen over Fha44-3M2e. Therefore, we inactivated the gene coding for full-length FHA (*fhaB*) in BPZM2e by introducing a pFus2 derivative that contains an internal fragment of *fhaB* [24]. As pFus2 cannot replicate in *B. pertussis*, the integration of the plasmid into the *fhaB* gene is forced by homologous recombination, thereby interrupting this gene. However, an approximately 20-kDa N-terminal fragment may still be produced. The integrants were selected on BG blood agar containing 100 µg/ml streptomycin and 10 µg/ml gentamycin, and the resulting strain was named BPZM2e-ΔFHA.

**Figure 3:**
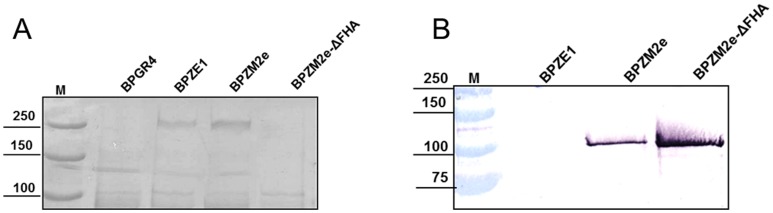
SDS-PAGE and Immunoblot analyses of culture supernatants from BPZM2e and BPZM2e-ΔFHA. Unconcentrated culture supernatants of the indicated strains were subjected to SDS-PAGE and Coomassie-blue staining (A) and Immunoblot analysis (B) using a monoclonal antibody to M2e. The molecular weight markers and their sizes in kDa are indicated on the left.

The absence of full length FHA in BPZM2e-ΔFHA was verified by SDS-PAGE and staining with Coomassie blue, and the presence of Fha44-3M2e in the culture supernatant was determined by immunoblot analysis. SDS-PAGE and Coomassie blue staining showed the absence of the 220-kDa protein, corresponding to FHA, in the culture supernatant of the BPZM2e-ΔFHA mutant strain, as well as in that of BPGR4, a known FHA-deficient strain, used as a control (Figure 3A). Immunoblot analyses of crude culture supernatants indicated that the FHA-deficient mutant produced at least as much Fha44-3M2e as the BPZM2e parent strain (Figure 3B). As expected, no immunoreactive band was detected in the culture supernatant of BPZE1.

### Lung Colonization and Immunogenicity of BPZM2e-ΔFHA

Many studies have indicated a crucial role of FHA in the colonization of the respiratory tract [25]–[28]. The ability of FHA-deficient mutants to colonize the murine respiratory tract is particularly impaired when pertussis toxin (PTX) is also absent or genetically inactivated [28], which is the case for all BPZE1 derivatives [5]. However, due to the presence of Fha44-3M2e, and/or an approximately 20-kDa N-terminal fragment of FHA that still might be produced instead of full-length FHA, it may be possible that BPZM2e-ΔFHA can still colonize sufficiently well to induce immune responses [27].

**Figure 4:**
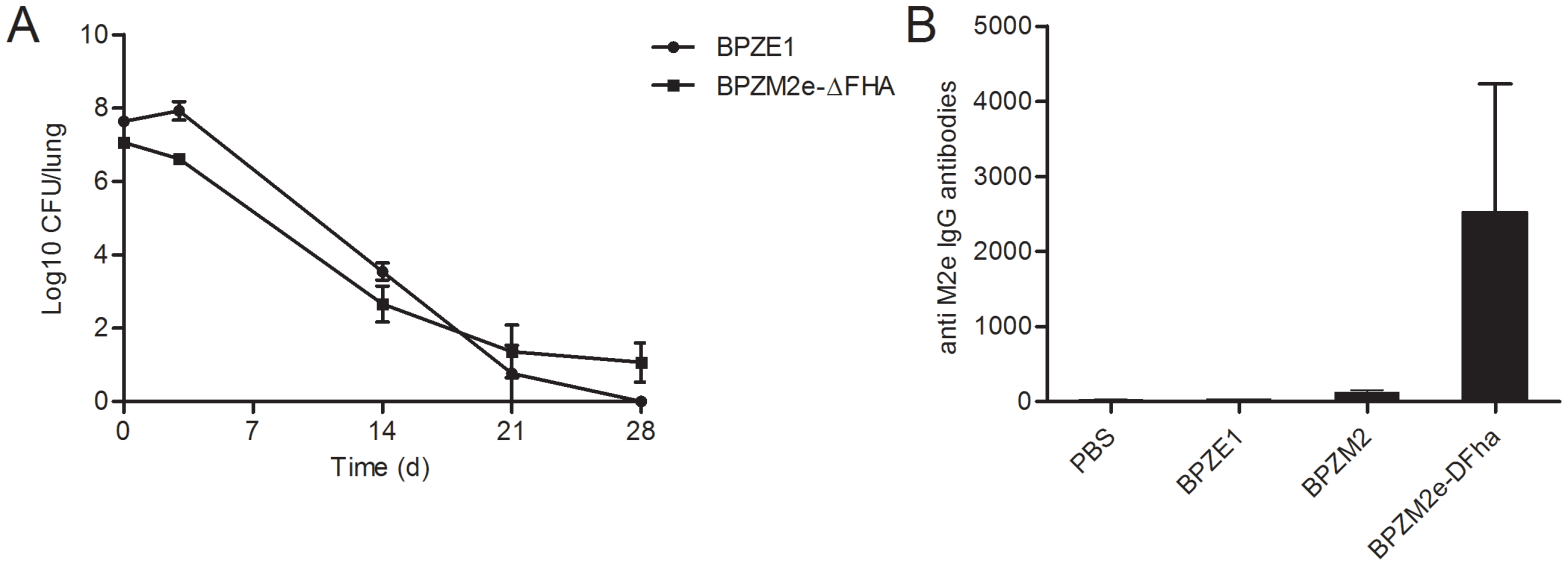
Colonization capacity and immunogenicity of BPZM2e-ΔFHA. (A) BALB/c mice were i.n. infected with 10^7 ^CFU of BPZE1 (black dots) or BPZM2e-ΔFHA (black squares), and the CFU in the lungs were quantified at the indicated time points. (B) BALB/c mice were i.n. immunized twice at a 4-week interval with 10^7 ^CFU of the indicated strains. Sera were collected two weeks after the last immunization, and serum IgG responses to M2e were determined by an M2e-peptide ELISA. The results are expressed as means ± standard errors. ***, *p*<0.001.

To investigate the colonization profile of the recombinant strain with genetically inactivated FHA, BALB/c mice were infected i.n. with 10^7^ CFU of BPZM2e-ΔFHA, and the bacterial load in the lungs was followed for up to 28 days. Both BPZE1 and BPZM2e-ΔFHA colonized the lungs and persisted in the lungs of the mice at similar levels, although at day 3 after inoculation slightly less BPZM2e-ΔFHA than BPZE1 was detected in the lungs of the mice (Figure 4A).

The immunogenicity of BPZM2e-ΔFHA was next evaluated after two i.n. administrations with a 4-week interval. The sera were collected 2 weeks after the last immunization, and the systemic anti-M2e antibody response was determined by peptide ELISA. BPZM2e-ΔFHA induced high levels of systemic IgG against M2e, whereas two administrations of BPZM2e, producing FHA, or BPZE1 did not result in a significant anti-M2e IgG response (Figure 4B). These observations indicate that the absence of FHA strongly increases the immune responses to M2e after i.n. immunization with recombinant BPZM2e-ΔFHA without affecting its lung colonization capacity.

### Heterologous i.n. Prime/boost Vaccination and Protection against Influenza Challenge

**Figure 5:**
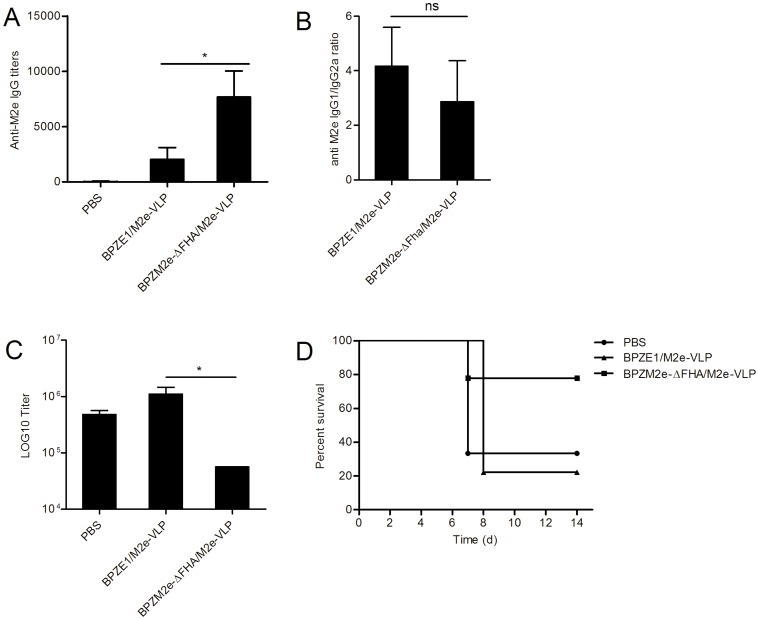
Anti-M2e IgG responses and protection against viral challenge after heterologous prime/boost vaccination. BALB/c mice were i.n. immunized with 10^7 ^CFU of BPZE1 or BPZM2e-ΔFHA as indicated and boosted i.n. 4 weeks later with 10 µg M2e-VLP. Negative control mice received PBS. Sera were collected 2 weeks after the last immunization, and serum IgG responses to M2e were determined by an M2e-peptide ELISA (A). IgG1 and IgG2a ratios (B) were determined for the groups primed with BPZE1 or BPZM2e-ΔFHA and boosted with M2e-VLP. (C) Groups of 3 BALB/c mice were i.n. primed with 10^7 ^CFU of BPZE1 or BPZM2e-ΔFHA and boosted 4 weeks later with 10 µg M2e-VLP. Two weeks after the last immunization the mice were challenged with 4 LD_50_ of influenza A virus, and the viral loads in the lungs were measured at 4 days post challenge by TCID_50_ quantification. The results are expressed as means of log10± standard errors. (D) Groups of 9 BALB/c mice were i.n. primed with 10^7 ^CFU of BPZE1 or BPZM2e-ΔFHA and boosted 4 weeks later with 10 µg M2e-VLP. Two weeks after the last immunization the mice were challenged with 4 LD_50_ of influenza A virus, and the percent of survival after challenge was monitored for up to 2 weeks. *, *p*<0.05; ns, not significant.

To investigate the priming effect of BPZM2e-ΔFHA, BALB/c mice were primed i.n. with 10^7^ CFU BPZM2e-ΔFHA or BPZE1 and boosted i.n. with M2e-VLP [20] in the absence of adjuvant. Systemic M2e-specific IgG responses were determined by ELISA two weeks after the last immunization. Mice primed with BPZM2e-ΔFHA and boosted with M2e-VLP mounted a significantly higher antibody titer than mice that had been primed with BPZE1 and subsequently received M2e-VLP. No anti-M2e antibodies were detectable in the control group of mice that had received twice PBS (Figure 5A). Priming with BPZM2e-ΔFHA did not modify the antibody isotype profile to M2e, compared to priming with BPZE1, as the IgG/IgG2a ratios were similar in both groups (Figure 5B).

Two weeks after the last immunization, the mice were challenged with a dose that corresponds to 4 LD_50_ of the mouse-adapted H3N2 influenza A virus X47. The M2e sequence of the X47 strain differs from that of the sequence used in BPZM2eΔFHA by one amino acid, Ser-12 to Asn, in addition to the two cysteine to serine changes. Three mice per group were used to determine the viral titers in the lung homogenates at day 4 after challenge. The mice primed with BPZM2e-ΔFHA and boosted with M2e-VLP had significantly lower viral loads in the lungs than BPZE1-primed mice boosted with M2e-VLP and than sham-vaccinated mice (Figure 5C). These results indicate that i.n. administration of BPZM2e-ΔFHA can efficiently prime for protective anti-viral immunity. When the mice were vaccinated once or twice with BPZM2e-ΔFHA without a M2e-VLP boost, no reduction in viral load was observed (data not shown), indicating that the M2e-VLP boost is necessary to reduce lung virus replication.

To investigate the effect of the heterologous prime-boost vaccination on mouse survival after viral challenge, we followed the mortality of BPZM2e-ΔFHA-primed and M2e-VLP-boosted mice for up to two weeks. The percent of survival was significantly higher in the group that had received BPZM2e-ΔFHA and M2e-VLP compared to the negative control group and the group that had received BPZE1 followed by M2e-VLP (Figure 5D). Again, BPZM2e-ΔFHA alone given once or twice did not result in a significant effect on survival (data not shown), indicating that, as for the effect on the viral load, the M2e-VLP boost was important to protect against mortality.

### Protection against *B. pertussis* Challenge

**Figure 6:**
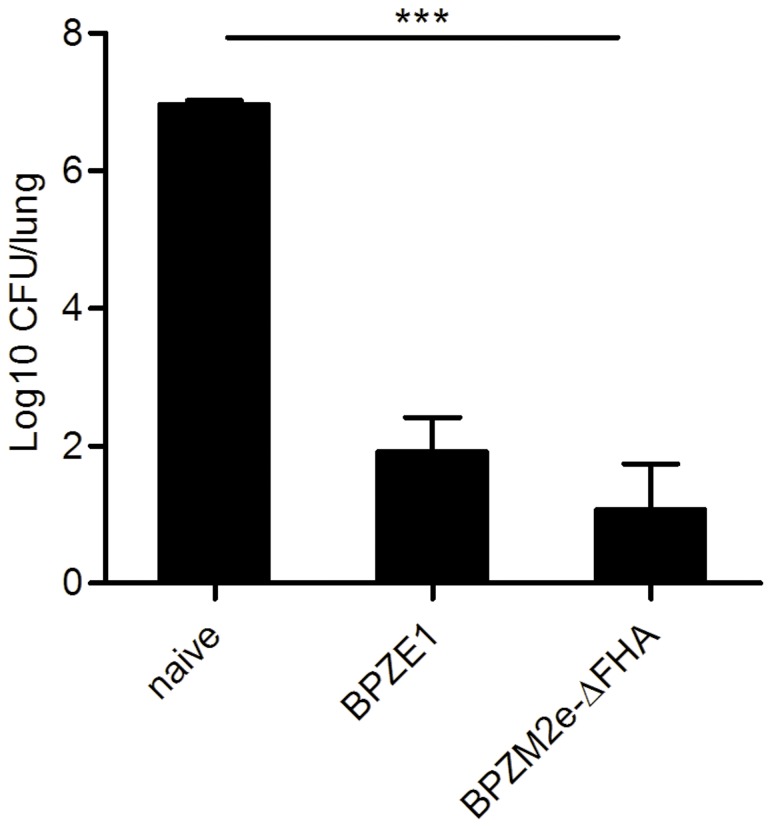
Protection against *B. pertussis* challenge. Groups of 5 BALB/c mice were i.n. immunized with 10^5 ^CFU of BPZE1 or BPZM2e-ΔFHA and challenged 4 weeks later with 10^6 ^CFU of BPSM. Seven days after infection, the mice were sacrificed and bacterial loads in the lungs were determined and are expressed as CFU/lung. The results are expressed as means ± standard errors. ***, *p*<0.001.

Since our general objective is to construct BPZE1 derivatives that are able to protect against both pertussis and other respiratory infections, it was important to verify that the genetic alterations in BPZM2e-ΔFHA did not alter its protective properties against pertussis. To evaluate the protective effect of BPZM2e-ΔFHA against *B. pertussis*, BALB/c mice were immunized i.n. with 10^5^ CFU BPZE1 [29] or BPZM2e-ΔFHA and challenged 4 weeks later with 10^6^ CFU of virulent *B. pertussis* BPSM. Seven days after challenge, the bacterial load was quantified in the lungs. As shown in Figure 6, a single administration of BPZM2e-ΔFHA or BPZE1 provided similar levels of protection against BPSM challenge. These data indicate that the inactivation of full-length FHA and the production of Fha44-3M2e do not interfere with the protective potential of BPZE1 against challenge with virulent *B. pertussis*.

## Discussion

Influenza remains a major public health problem world-wide and causes serious illness in 3 to 5 million people, including 200,000 to 500,000 deaths at each flu season. It is the seventh leading cause of death in the United States [30]. Particularly the elderly, immunocompromized and the very young are at risk for severe influenza and account for more than 90% of all influenza-related deaths. In addition to seasonal influenza, pandemic influenza constitutes a constant public health threat, and due to the modern lifestyle of human society, there is an elevated risk for the emergence of new virus reassortants with increased virulence. Antiviral drugs may be an effective way of treating the disease. However, their effectiveness is limited by the need for early initiation of treatment in the course of infection [31] and by the risk of resistance to the drugs [32]. Vaccination is the most cost-effective measure to control the disease.

Current influenza vaccines are based on three virus strains, used either as inactivated or live attenuated whole viruses, as split virus vaccines or as subunit vaccines and comprise essentially the immunodominant antigens hemagglutinin and neuraminidase. However, the antigenic make-up of the virus constantly changes through two mechanisms called “antigenic drift” and “antigenic shift” [33]. As a consequence, the vaccines have poor clinical efficacy against viral strains that do not match the vaccine strain [34], and vaccine types have to be adapted nearly every year to the viral types that are predicted to circulate during the forthcoming winter season.

The search for a universal influenza vaccine has thus been an endeavour for decades now. M2e is the extracellular domain of the influenza virus matrix protein M2, and its amino acid sequence is remarkably conserved among viral strains [18]. Although present in only small amounts on the viral particles, it is abundantly expressed on the surface of infected cells, and antibodies to this peptide have been reported to limit viral replication and to protect from challenge with various viral strains. It has therefore been proposed as a universal influenza vaccine candidate [19].

In this study, we used recombinant derivatives of the novel live attenuated *B. pertussis* vaccine candidate BPZE1 to present M2e to the respiratory mucosa in order to induce anti-M2e antibodies by i.n. administration. In addition to logistic advantages, this needle-free approach is also expected to induce broader cross-protection against heterologous virus strains, compared to parenteral vaccination [35]. Although due to its strong sequence conservation, this may be less an issue when M2e is used as an antigen. Nevertheless, some sequence variations in M2e have been detected among strains. BPZE1 has already been used in experimental animal models to present heterologous antigens [16], [17], including M2e [17]. Previous work has used full-length FHA or the autotransporter BrkA to surface display the heterologous antigen on BPZE1. However, the insertion of 3 copies of M2e into full-length FHA induced only a modest serum antibody response to the antigen, even after repeated administrations of the recombinant strain. In addition, the insertion of three copies of M2e into FHA significantly affected FHA secretion into the extracellular milieu and the ability of the recombinant strain to colonize the mouse lungs [17]. Decreasing the numbers of M2e copies in FHA improved FHA secretion and lung colonization, but at the expense of M2e immunogenicity.

In this study we used Fha44, a truncated form of FHA as a genetic fusion partner for M2e. Fha44 is more efficiently secreted than FHA in *B. pertussis* [22]. The fusion of 3 copies of M2e to Fha44 did not appear to impair the secretion of the Fha44-3M2e chimera, as the recombinant protein could readily be detected in crude BPZM2e culture supernatants. Also, the general fitness of the BPZE1-derivative, and its ability to colonize the mouse respiratory tract were not affected by the fusion of 3 copies of M2e to Fha44. Nevertheless, even repeated administrations of BPZM2e did not result in a detectable antibody response to M2e, nor did they prime for an anti-M2e antibody response (data not shown).

However, when the chromosomal gene of the full-length FHA was inactivated by a mutational insertion, the resulting BPZM2e-ΔFHA strain induced strong antibody titers against M2e after i.n. administration. The insertional inactivation probably resulted in the production of a small truncated N-terminal fragment of FHA rather than in its total absence. Importantly, the inactivation by plasmid insertion was stable, as verified after repeated in vitro culturing and after in vivo passaging (data not shown).

A single i.n. administration of BPZM2e-ΔFHA also primed anti-M2e responses, which could be strongly boosted by an i.n. administration of M2e-VLP. This heterologous prime/boost regimen resulted in significant protection against lethal challenge with influenza A virus and in decreased viral load after challenge, even though the M2e sequence of the challenge strain differed by three amino acids from that in BPZM2e-ΔFHA. This slight sequence variation did thus not hamper the protective effect of the prime-boost regimen used here.

M2e has been tested before as a nasal vaccine and found to induce protection by that route. However, protection was achieved after 3 doses of vaccine and preferably in the presence of a potent mucosal adjuvant [19], [20], [36]. In the heterologous prime/boost strategy described here, a single i.n. administration of the M2e-VLP provided protection when the mice were primed with BPZM2e-ΔFHA. As no nasal adjuvant has yet been approved for human use, and as BPZE1 has now successfully concluded a phase I safety trial in humans (Thorstensson et al., in preparation), this strategy may be a promising approach for needle-free delivery of universal influenza vaccines.

BPZE1 was originally designed as a priming vaccine against pertussis for a strategy in which the current acellular vaccines would serve as booster vaccines to improve protection and lengthen vaccine efficacy. The results shown here support the concept that recombinant BPZE1 derivatives can be used as priming vaccines for multiple respiratory infections by using the FHA secretion machinery to surface expose or secrete heterologous protective antigenic peptides. Interestingly, Fha44-3M2e was only immunogenic when delivered i.n. by a BPZE1 derivative that did not produce full-length FHA. Although the mechanism of this effect has not been definitively established yet, it may be related to the immuno-regulatory properties of full-length FHA. FHA has been described to inhibit IL-12 production and to stimulate IL-10 production by dendritic cells, resulting in the induction of regulatory T cells [37]. Alternatively, since FHA forms multimeric bundles [38], the presence of full-length FHA in BPZM2e may potentially mask the viral epitope on Fha44-3M2e. Finally, we cannot exclude that the increased anti-M2e responses may be related to a potentially slightly higher Fha44-3M2e production in BPZM2e-ΔFHA compared to BPZM2e.

In the context of inactive PTX, FHA is important to establish bacterial colonization in the respiratory tract [28], which, in turn, is essential for the induction of protective immunity against pertussis [5]. It was therefore important to observe that the inactivation of the FHA gene in BPZM2e-ΔFHA does not impair the colonization, nor the ability of the strain to protect against virulent *B. pertussis* challenge. In BPZM2e-ΔFHA, Fha44 could thus functionally replace full-length FHA for colonization and induction of anti-*B. pertussis* immunity.

Although M2e has been documented by several studies as a promising universal vaccine candidate against influenza, other conserved epitopes have also recently been identified. Studies on the B cell repertoire of subjects infected with the 2009 pandemic H1N1 influenza virus have uncovered broadly cross-reactive epitopes in the hemagglutinin, that can neutralize a variety of influenza A virus subtypes *in vitro* [39]. In addition to antibodies, cross-reactive CD4^+^ and CD8^+^ T cells may also play an important role in protection against influenza [40]. Multimeric vaccine constructions comprising arrays of several protective and cross-reactive T and B cell epitopes may thus be an interesting option to pursue [41], [42] and to produce in recombinant BPZE1 derivatives.

## Materials and Methods

### Ethics Statement

All animal experiments were performed following the guidelines of the Institut Pasteur de Lille animal study board, which conforms to the Amsterdam Protocol on animal protection and welfare, and Directive 86/609/EEC on the Protection of Animals Used for Experimental and Other Scientific Purposes, updated in the Council of Europe’s Appendix A (http://conventions.coe.int/Treaty/EN/Treaties/PDF/123-Arev.pdf). The animal work also complied with the French law (n° 87–848 dated 19-10-1987) and the European Communities Amendment of Cruelty to Animals Act 1976. All manipulations involving animals were carried out by qualified personnel. The animal house was placed under the direct control of the Institut Pasteur de Lille director who is the “designated responsible person” under French law. The study has been approved by Ethical Committee for experiments on animals of the region Nord-Pas-de-Calais (approval number AF 03/2009).

Influenza A virus challenge experiments of immunized BALB/c mice were performed at the Department for Molecular Biomedical Research, VIB, Ghent, Belgium in a biosafety level 2 contained animal house. Influenza virus challenge protocols were authorized by the Institutional Ethics Committee on Experimental Animals (permit number LA1400091). All efforts were made to reduce suffering of animals.

### Bacterial Strains and Growths Conditions

The *B. pertussis* strains used in this study are listed in Table 1. They were grown on Bordet-Gengou agar (Difco, Detroit, Mich.) supplemented with 1% glycerol, 20% defibrinated sheep blood, and 100 µg/ml streptomycin (Sigma Chemical Co., St Louis, Mo.) at 37°C for 72 h. Liquid cultures of *B. pertussis* were grown as described previously [43] in Stainer-Scholte medium [44] containing 1 g/liter heptakis (2,6-di-o-methyl) β-cyclodextrin (Sigma).

**Table 1:** *B. pertussis* strains used in this study.

Strain	Description	Reference
BPSM	Sm^R^ and Nal^R^ derivative of *B. pertussis* Tohama I	24
BPZE1	Attenuated vaccine strain derivative of BPSM	5
BPGR4	FHA-deficient derivative of BPSM	50
BPZM2e	BPZE1 derivative producing Fha44-(M2e)_3_	This work
BPZM2e-ΔFHA	FHA-deficient BPZMe2 derivative	This work

### Construction of Plasmids and Recombinant BPZE1 Strains

The Fha44-3M2e-coding DNA was inserted into the *dnt* locus of BPZE1 by allelic exchange, using a plasmid derived from pJQmp200rpsL12 [45] and containing up- and downstream regions of the *dnt* gene, as described by Mielcarek et al. [5] for the deletion of the *dnt* gene in BPZE1. This plasmid was named pJQdntUPLO. To construct pXR1-Fha44, the TCTAGA *Xba*I site of pJQdntUPLO was first changed to TCCAGA using QuickChangeIl® XL (Stratagen) according to the manufacturer’s instructions. This yielded pXR1. A synthetic gene coding for the 5′ part of the *fhaB* gene was purchased from Eurogentec (Liège, Belgium). This gene, named *fha44c*, contains 2,583 bp coding for amino acids 1 to 861 of FhaB, the precursor of FHA (from nucleotide position 253 to 2,835, GenBank M60351.1), except for four silent changes (G354C, C864G, G2,331C and A2,556G), a 27-bp multiple cloning site with the sequence 5′-CTTAAGACGCGTCATATGGGCGGCCGC-3′ and two TGA termination codons. This sequence was provided in a plasmid named pUC57-Fha44_c_, which was digested with *Xho*I and *Xba*I. The fragment corresponding to *fha44c* was inserted into *Xho*I/*Xba*I-digested pXR1, yielding pXR1-Fha44.

The sequence coding for 3 copies of the M2e peptide was amplified by PCR from the pGA4-3M2e (purchased from Geneart AG), containing the coding information for three tandem copies of M2e. Each M2e copy in pGA4-3M2e is separated by SGSGGSGGS and cysteine codons at positions 17 and 19 in M2e were replaced by serine. Oligonucleotides 5′-ACGCGTGTGGAAACTCCTATCCG-3′ and 5′-CATATGGCCGCCAGAGCCGCTATCAGAGCTATCGTT-3′ were used as primers. The amplified DNA was then inserted into pCRII-TOPO (Invitrogen). A 273-bp fragment obtained after *Mlu*I/*Nde*I digestion was inserted into *Mlu*I/*Nde*I-digested pXR1-Fha44 to yield pHKG3, which was then introduced into *E. coli* SM10 [46] by transformation. The resulting recombinant *E. coli* SM10 was then conjugated with BPZE1. Two successive homologous recombination events were selected as described [47]. The ex-conjugants were screened by PCR to ensure that the hybrid gene was correctly inserted in the *B. pertussis* chromosome. The recombinant BPZE1 strain was named BPZM2e. To construct the FHA-deficient BPZM2e derivative BPZM2e-ΔFHA, the FHA-encoding gene was inactivated using the integration vector pFUS2, as previously described [24].

### Protein Analysis and Immunodetection of Fha44-3M2e

For the detection of Fha44-3M2e, BPZM2e and BPZM2e-ΔFHA, as well as BPZE1 were grown for 48 hours in 10 ml Stainer-Scholte medium supplemented with 100 µg/ml Streptomycin. The cells were then centrifuged for 15 minutes at 4,000×*g*. Supernatant was collected, and the cells were resuspended in 1 ml PBS. Cells were broken using a French Pressure cell, and the lysates were clarified by centrifugation at 4,000×*g* for 15 min at 4°C. The culture supernatants and the whole cell extracts were analyzed by SDS/polyacrylamide gel electrophoresis on 10% polyacrylamide gels as described by Laemmli [48] and by immunoblot analyses using an anti-M2e monoclonal antibody [19] at a 1∶3,000 dilution.

### Mouse Infection

BALB/c mice were obtained from Charles River (l’Abresle, France) and maintained under specific pathogen-free conditions in the animal facilities of the Institut Pasteur de Lille. For *B. pertussis* infection, 6 week-old BALB/c mice were lightly sedated by intraperitoneal injection with an anesthetic cocktail (ketamine+atropine+valium) before i.n. infection with 20 µl PBS containing 10^6^ or 10^7^ colony-forming units (CFU) of *B. pertussis* BPZE1, BPZM2e, BPZM2e-ΔFHA or BPSM, as previously described [5]. The mice were sacrificed at selected time points after i.n. administration, and their lungs were harvested, homogenized in PBS and plated in serial dilutions onto BG-blood agar to count CFUs after incubation at 37°C for three to four days, as described [5], [8]. When indicated, the mice were boosted i.n. 4 weeks after priming with 10 µg M2e-VLP without adjuvant [36].

### Antibody Detection

96-well plates were coated overnight at 4°C with 2 µg/ml of the synthetic peptide M2e with the following sequence: GGSLLTEVETPIRNEWGSRSNDSSDGG, washed with PBS containing 0.05% Tween-20 (PBST) and then blocked with 100 µl/well of blocking buffer (2% BSA in PBST) for 1 h at 37°C. After three washes, 100 µl of serially diluted sera was added to the wells and incubated for 2 h at 37°C. After another set of three washes, the plates were incubated for 1 h at 37°C with 100 µl of horseradish-peroxidase-labeled goat anti-mouse IgG, IgG1 or IgG2a (Southern Biotech) diluted respectively at 1∶4,000, 1∶1,000 or 1∶2,000 in PBST. Following five washes, the plates were incubated with 100 µl of HRP Substrate TMB solution (Interchim) for 30 min at room temperature. The reaction was stopped by the addition of 50 µl of 1 M H_3_PO_4_. The optical density (OD) was measured with a Biokinetic reader EL/340 microplate at 450 nm. Titers were determined as the highest serum dilution that had an optical density reading more than twice that of the negative control serum.

### Viral Challenge

Two weeks after the last immunization the mice were challenged. Fifty microliters of a lethal dose (4 LD50, corresponding to 2.5 × 10^3^ TCID50) of a mouse-adapted X47 virus (A/Victoria/3/75 (H3N2) ×PR/8) [19] was administered i.n. to mice lightly anesthetized by isoflurane. Mortality was monitored on a daily basis for 2 weeks. Morbidity was assessed by body weight measurement.

Four days after challenge, 3 or 5 mice from each group were sacrificed by cervical dislocation. Lungs were removed aseptically and snap-frozen in liquid nitrogen. Lung extracts were made by homogenizing the frozen tissue in a precooled mortar and resuspending the homogenate in 5 ml of sterile, ice-cold PBS. The extracts were transferred to centrifuge tubes, and cell debris was removed by centrifugation for 5 min at 400×*g* and 4°C. The cleared lung extracts were stored at −80°C. Viral titers were determined in triplicate by titration on MDCK cells. Cell monolayers were infected for 1 h with 50 µl of serial 1∶10 dilutions of the lung extracts in a 96-well plate in serum-free DMEM medium (Invitrogen, Merelbeke, Belgium) supplemented with penicillin and streptomycin. Following inoculation, the supernatant was replaced by serum-free DMEM medium containing 2 µg/ml trypsin. Endpoint virus titers were determined after 4 days, as described [49], by interpolating the dilution that infected 50% of the wells, as assayed by hemagglutination of chicken red blood cells.

### Statistical Analyses

The results were analyzed using the unpaired Student's *t*-test (GraphPad Prism Program). Differences were considered significant at p<0.05.
